# New Horizons in Transcatheter Aortic Valve Replacement: Expectations and Preparations

**DOI:** 10.3390/jcm15093479

**Published:** 2026-05-01

**Authors:** Haleema Nawaz, Abdellaziz Dahou, Tariq Ahmad

**Affiliations:** 1 Department of Internal Medicine, Geisinger Wyoming Valley Medical Centre, Wilkes-Barre, PA 18711, USA; hnawaz1@geisinger.edu; 2 Department of Cardiology, Geisinger Wyoming Valley Medical Centre, Wilkes-Barre, PA 18711, USA; adahou@geisinger.edu

**Keywords:** transcatheter aortic valve replacement, aortic stenosis, valve-in-valve TAVR, prosthesis–patient mismatch, coronary obstruction, structural heart disease

## Abstract

Transcatheter aortic valve replacement (TAVR) has transformed the management of severe aortic stenosis and is now widely used across a broad spectrum of surgical risk. With expanding indications and increasing use in younger patients, contemporary practice increasingly emphasizes lifetime management of aortic valve disease, a shift further supported by recent developments including findings from the EARLY TAVR trial and the May 2025 U.S. Food and Drug Administration approval of TAVR for asymptomatic severe aortic stenosis. This narrative review summarizes recent developments in TAVR, including advances in device technology, procedural techniques, and patient selection. Focus is placed on the importance of optimal first valve selection, prevention of prosthesis–patient mismatch (PPM), and planning for future reintervention such as valve-in-valve (ViV) TAVR. Emerging procedural strategies including bioprosthetic valve fracture and leaflet modification techniques have expanded treatment options for patients at risk of elevated gradients or coronary obstruction. The review also highlights evolving approaches to TAVR in complex clinical scenarios and discusses future directions in device design and imaging-based procedural planning. As TAVR continues to evolve, careful procedural planning and multidisciplinary heart team collaboration remain essential to optimizing long-term outcomes.

## 1. Methodology

This narrative review was conducted to provide a comprehensive and contemporary overview of transcatheter aortic valve replacement (TAVR), with a focus on device evolution, procedural strategies, and lifetime management considerations. A targeted literature search was performed using electronic databases including PubMed/MEDLINE, Embase, and Google Scholar for articles published up to 2025. Search terms included combinations of keywords such as “transcatheter aortic valve replacement,” “TAVR,” “TAVI,” “prosthesis–patient mismatch,” “valve-in-valve,” “coronary obstruction,” “BASILICA,” “leaflet modification,” “bioprosthetic valve fracture,” and “coronary protection strategies.”

Articles were selected based on relevance to the scope of the review, with emphasis on randomized controlled trials, large observational studies, registry data, guideline documents, and high-impact review articles. Seminal studies and landmark trials were prioritized to provide historical context, while recent publications were included to reflect current practice and evolving evidence. Case reports and small series were selectively included where they described novel procedural techniques or emerging technologies not yet supported by large-scale data. Reference lists of selected articles were manually screened to identify additional relevant studies. Only English-language publications were included. Given the narrative nature of this review, no formal systematic review protocol or quantitative synthesis was performed.

## 2. Brief History of Transcatheter Aortic Valve Replacement

Transcatheter aortic valve replacement (TAVR) represents one of the most transformative innovations in cardiovascular medicine in the 21st century, fundamentally altering the management of patients with aortic stenosis. What is now a major therapeutic advancement began modestly as a case series of animal implantations presented at the European Society of Cardiology Congress in 1992, followed by the first in-human implantation performed by Alain G. Cribrier, MD (1945–2024), in Rouen, France, in 2002. Over the subsequent two decades, TAVR has undergone a remarkable evolution from a disruptive and experimental concept to an established standard of care. This odyssey exemplifies a sustained commitment to rapid technological advancement through collaborative, science-driven efforts, with optimal patient care as the central objective. The most recent milestone in this progression is the U.S. Food and Drug Administration approval of TAVR for asymptomatic, low-risk patients with aortic stenosis in May 2025, following the results of the EARLY TAVR trial [1,2].

Beyond the expansion of its clinical indications, TAVR has undergone substantial refinement in device technology and procedural techniques. Notable advances include the development of lower-profile delivery systems that obviate the need for surgical cutdowns, leaflet calcium-blocking technologies, enhanced skirt designs to mitigate paravalvular regurgitation, modifications in valve frame architecture to facilitate coronary re-access, and improved delivery catheter designs that enable smoother navigation through calcified and tortuous vascular anatomies. In addition, adjunctive technologies such as shockwave intravascular lithotripsy have further expanded the feasibility of transfemoral TAVR in patients with heavily calcified peripheral arterial disease.

## 3. Near Future Expectations

**Figure 1:**
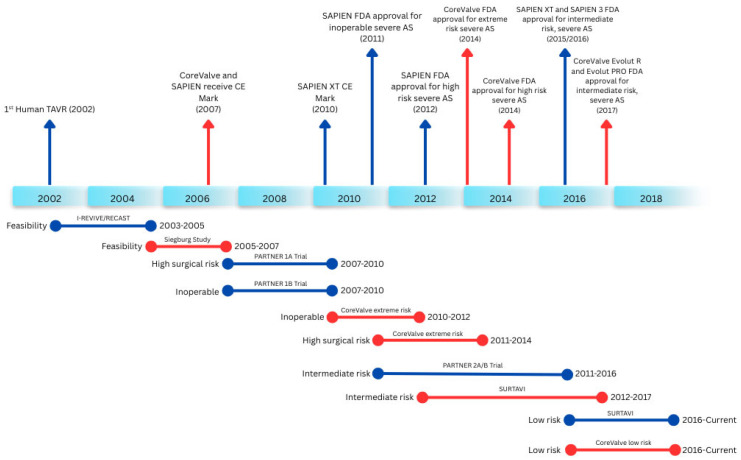
Timeline of major transcatheter aortic valve replacement (TAVR) trials and approvals. CE, Conformité Européenne; FDA, Food and Drug Administration; AS, aortic stenosis; I-REVIVE, Initial Registry of EndoVascular Implantation of Valves in Europe; RECAST, Registry of Endovascular Critical Aortic Stenosis Treatment; PARTNER, Placement of AoRTic traNscathetER valves; SURTAVI, Surgical or Transcatheter Aortic Valve Replacement in Intermediate-Risk Patients. Adapted from [4].

**Figure 2:**
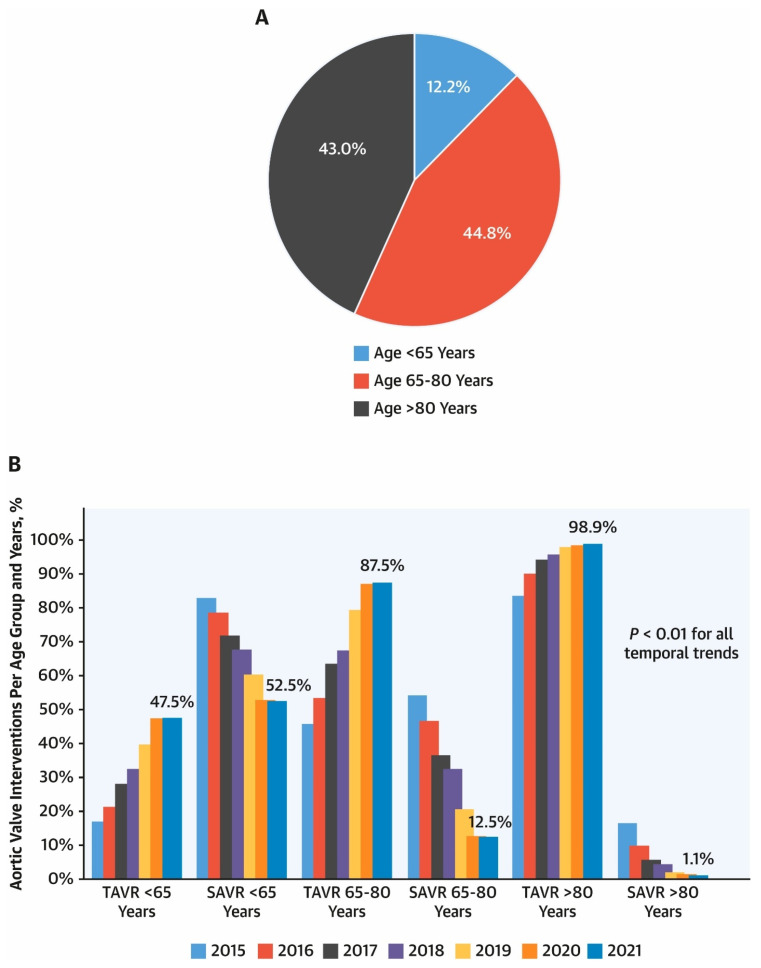
Aortic Valve Interventions for Isolated Aortic Stenosis Stratified According to Age. (**A**) Total aortic valve interventions (2015–2021) for isolated aortic stenosis stratified by age groups. Illustration of the total number of aortic valve interventions (transcatheter aortic valve replacement [TAVR] or surgical aortic valve replacement [SAVR]) for the entire study period 2015 to 2021 (N = 142,953) for isolated aortic stenosis stratified according to the American Heart Association/American College of Cardiology guideline-recommended age groups. (**B**) Trends in TAVR vs. SAVR stratified by guideline-recommended age groups. Illustration of temporal trends in TAVR vs. SAVR utilization. Despite SAVR being recommended by American Heart Association/American College of Cardiology guidelines for patients < 65 years old, volumes are nearly equal between SAVR and TAVR for younger patients by 2021. Reproduced with permission from [3].

In parallel with these technological and procedural advancements, the utilization of TAVR has increased rapidly across a broad spectrum of patients (Figure 1), including a growing proportion of younger individuals. Notably, TAVR use among patients younger than 65 years has risen to near parity with surgical aortic valve replacement (SAVR). A study using the Vizient database, which captures data from approximately 800 U.S. academic medical centres, demonstrated that by 2021, TAVR utilization in patients under 65 years of age had approached rates comparable to SAVR (Figure 2) [3].

Similarly, an analysis of temporal trends in TAVR and SAVR using data from the Northern New England Cardiovascular Disease Study Group (NNECDSG) Consortium, which included patients undergoing isolated aortic valve interventions and excluded those requiring concomitant or emergent procedures, those with endocarditis, and those with prior aortic valve replacement, showed that more than 50% of patients younger than 65 years underwent TAVR for treatment of aortic stenosis in 2022, compared with only 19% in 2016 [5].

Given these trends and the increasing use of TAVR in patients with an anticipated life expectancy of 10–15 years, it is foreseeable that a substantial number of patients will require either TAVR explantation or valve-in-valve (ViV) TAVR in the coming years.

In parallel, current trends indicate an increasing preference for bioprosthetic valves in patients undergoing surgical aortic valve replacement (SAVR) across all age groups, which is expected to further increase the future demand for TAVR following SAVR [6]. Yohei Ohno et al. estimated that approximately 15% of TAVR procedures performed in the United States will be valve-in-valve interventions by 2035 [7]. Surgical and transcatheter valves incorporating RESILIA tissue, which is designed to mitigate leaflet calcification, have been reported to demonstrate a lower incidence of structural valve deterioration requiring repeat valvular intervention [8].

In contrast, the Trifecta surgical valve was withdrawn from the market in February 2023 because of reports indicating an increased risk of structural valve deterioration compared with other available surgical bioprosthetic valves [9]. The Trifecta valve was more frequently implanted in patients with smaller annuli, owing to its externally mounted leaflet design that enabled a relatively larger predicted effective orifice area. The INSPIRIS RESILIA surgical valve incorporates an expansion zone within its frame that is intended to facilitate valve expansion during valve-in-valve TAVR procedures without the need for surgical valve frame fracture [10].

Patient–prosthesis mismatch (PPM) is a critical consideration in both initial valve selection and planning for valve-in-valve (ViV) TAVR [6]. The primary goal of aortic valve intervention for severe native valve dysfunction is to replace the diseased valve with a prosthesis that restores normal hemodynamics without residual stenosis or regurgitation. Despite this objective, a substantial proportion of prosthetic valves are associated with elevated residual transvalvular gradients due to PPM. PPM is classified as moderate when the indexed effective orifice area (EOA) is 0.65–0.85 cm^2^/m^2^ and severe when the indexed EOA is less than 0.65 cm^2^/m^2^.

**Figure 3:**
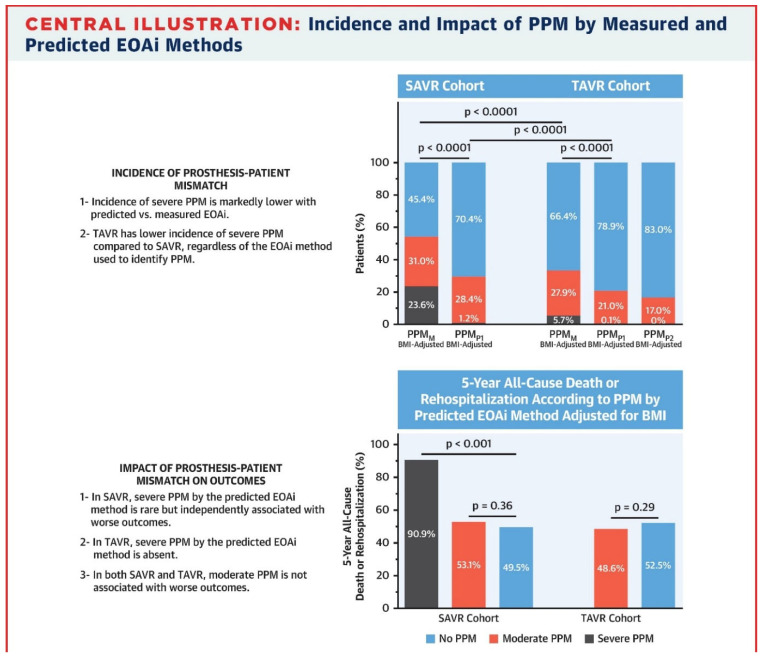
Incidence and Impact of PPM by Measured and Predicted EOAi Methods. Adapted from [11].

**Figure 4:**
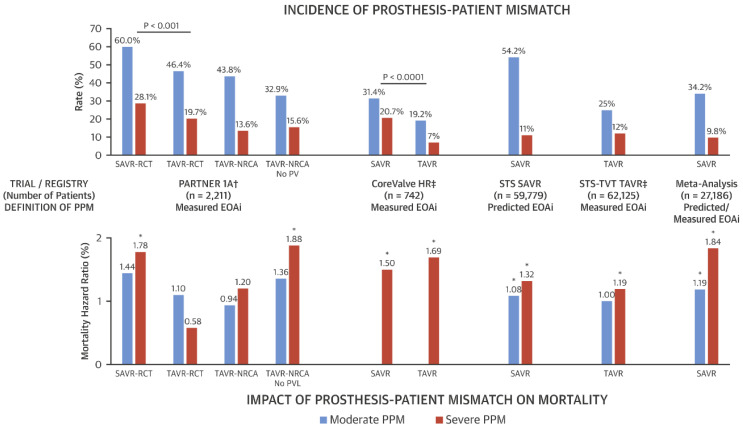
Incidence and Impact on Mortality of PPM Following AVR. Incidence of moderate and severe PPM, as well as the association of PPM with mortality following AVR, are shown. * Statistically significant; ^†^ 2-year mortality; ^‡^ 1-year mortality. CoreValve HR = CoreValve Pivotal High-Risk trial; EOAi = indexed effective orifice area; NRCA = Non-Randomized Continued Access registry; PARTNER 1A = Placement of AoRTic TraNscathetER Valve 1A trial; PPM = prosthesis–patient mismatch; PVL = paravalvular leak; RCT = randomized controlled trial; SAVR = surgical aortic valve replacement; STS = Society of Thoracic Surgeons; STS-TVT = STS/ACC TVT Registry; TAVR = transcatheter aortic valve replacement; TVT = transcatheter valve therapy. Reproduced with permission from [16].

PPM occurs more frequently following surgical aortic valve replacement (SAVR) than TAVR and has been associated with an increased risk of mortality [11,12] (Figure 3 and Figure 4). Younger patients and female patients with relatively small aortic annuli are more likely to receive surgical valves smaller than 23 mm, which contributes to a higher incidence of PPM [13]. The use of computed tomography-based TAVR sizing to guide cardiothoracic surgeons in selecting appropriately sized bioprosthetic valves has been shown to significantly reduce the risk of PPM and decrease interoperator variability [14,15].

Aortic root enlargement to accommodate implantation of a larger surgical valve is another strategy that reduces the risk of PPM. However, this approach is utilized in only approximately 0.6% of cases and is predominantly performed at high-volume comprehensive valve centers [14]. Root enlargement may also play an important role in preventing valve deformation during surgical implantation [17], thereby improving valve hemodynamics through larger effective orifice areas, reducing the incidence of PPM, and potentially mitigating the risk of early structural valve deterioration.

The SMART trial [18] compared the self-expanding Evolut PRO, PRO+, and FX valves (Medtronic) with the balloon-expandable SAPIEN 3 and SAPIEN 3 Ultra valves (Edwards Lifesciences) in 716 low- to intermediate-risk patients with symptomatic severe aortic stenosis. The study population had a mean age of 80 years, and 87% were women. On preprocedural computed tomography planning, mean aortic annular area was similar between the self-expanding and balloon-expandable valve groups at 380.9 mm^2^ and 382.8 mm^2^, respectively, with corresponding mean annular perimeters of 70.3 mm and 70.4 mm.

At 2-year follow-up, there were no significant differences between treatment groups with respect to all-cause mortality, cardiovascular mortality, heart failure rehospitalization, or aortic valve reintervention. However, the balloon-expandable valve group demonstrated significantly higher residual transvalvular gradients compared with the self-expanding valve group. Mean gradients greater than 20 mmHg were observed in 42.2% of patients receiving balloon-expandable valves versus 4.7% in the self-expanding valve group (*p* < 0.001), while mean gradients greater than 25 mmHg occurred in 21.7% versus 2.4%, respectively (*p* < 0.001). Correspondingly, effective orifice area was smaller in the balloon-expandable valve group at 1.51 cm^2^ compared with 1.93 cm^2^ in the self-expanding valve group. Balloon-expandable valve recipients were also more likely to experience severe prosthesis–patient mismatch through 1 year, as defined by VARC-3 criteria (*p* < 0.001).

Prosthetic valve thrombosis occurred more frequently in the balloon-expandable valve group, affecting 15 patients compared with 3 patients in the self-expanding valve group (*p* = 0.005). Among these cases, 28% were classified as late events occurring between 30 days and 1 year, while 56% were very late events occurring beyond 1 year. Five episodes of valve thrombosis were clinically apparent and were associated with two myocardial infarctions, one stroke, one death, and one additional embolic event.

Supporting the potential long-term implications of residual gradients, data from the Australian National Echocardiography Database involving more than 6000 patients who underwent SAVR or TAVR demonstrated that elevated mean transvalvular gradients are associated with reduced survival, although this effect does not become apparent until approximately 3 years after valve replacement [19]. Similarly, prior data on prosthesis–patient mismatch have shown up to a 25% increase in mortality at 5 years [12], raising concern that the hemodynamic disparities observed between treatment arms in the SMART trial may ultimately translate into clinically meaningful differences over longer follow-up.

It is important to contextualize these findings with results from the RHEIA trial [20], which compared TAVR using the SAPIEN 3 valve with surgical aortic valve replacement in women with small aortic annuli. In RHEIA, the incidence of residual high transvalvular gradients greater than 20 mmHg was significantly lower than that observed in SMART, occurring in 4.7% of SAVR patients and 11.7% of TAVR patients (*p* = 0.039). Rates of severe prosthesis–patient mismatch were similarly low at 3.6% for SAVR and 4.7% for TAVR, with no statistically significant difference between groups.

The lower incidence of residual transvalvular gradients in the RHEIA trial is possibly due to a relatively larger annular area in the TAVR group compared to the BEV group in the SMART trial (403.7 ± 63.1 mm^2^ versus 379.1 ± 33.9 mm^2^), allowing placement of relatively larger valves (29 mm: 4.7% versus 0%; 26 mm: 27% versus 1.9%; 23 mm: 63.7% versus 88.8%; 20 mm: 4.7% versus 9.3%). Additionally, bicuspid valves were excluded in the RHEIA trial, whereas 4.9% of patients in the BEV group had bicuspid aortic valves, which can potentially lead to higher residual transvalvular gradients.

## 4. Selection of First Valve: A Decision with Future Implications

Selecting the optimal valve type in contemporary practice has become increasingly complex, given the expanding array of available devices, broadened indications, and a growing patient preference for less invasive therapies with faster postoperative recovery. At the same time, clinicians are increasingly tasked with incorporating a lifetime management framework into pre-procedural discussions to facilitate truly informed consent. With the patient placed at the center of this decision-making process, the overarching objective is to begin with the most appropriate initial valve choice.

An appropriately sized valve, particularly in patients with smaller aortic annuli defined as less than 430 mm^2^ by computed tomography, that incorporates a durable design to minimize the likelihood of reintervention, can be implanted using a safe and reproducible approach, and preserves future options for reintervention should be prioritized. Equally important is ensuring that this strategy aligns with patient goals and preferences. In practice, however, definitions of reasonable versus less favorable management strategies may vary based on the experience and expertise of the treating heart team, a concept supported by previously published observational data [5].

### Case Example

A 31-year-old woman with a history of severe bicuspid aortic valve stenosis previously underwent balloon aortic valvuloplasty at 19 years of age. The procedure was complicated by injury to the aorto-mitral continuity, necessitating open surgical aortic and mitral valve repair. She later presented to our practice with recurrent severe aortic stenosis during the third trimester of her first pregnancy. Transthoracic echocardiography demonstrated a peak aortic valve velocity of 4.8 m/s, a mean gradient of 55 mmHg, and an aortic valve area of 0.7 cm^2^. Given her advanced gestational age, she was managed conservatively and subsequently had an uncomplicated delivery.

**Figure 5:**
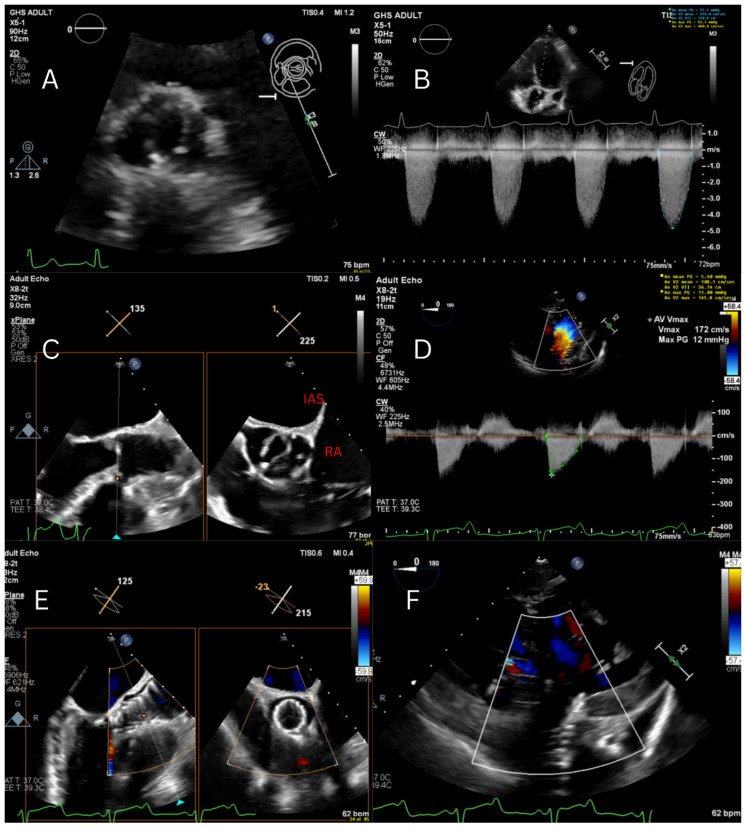
(**A**) Short axis view of the aortic valve on TTE showing bicuspid AV with fusion of the RCC and the NCC. (**B**) Aortic stenosis assessment by Doppler showing severe AS: Vmax 4.8 m/s, MG 55 mmHg, AVA 0.7 cm^2^. (**C**) TEE showing bicuspid AV with fusion of the RCC and the NCC (image on the right is inverted). (**D**,**E**) Immediately post-TAVR TEE result: AV MG 5 mmHg, DI 0.78, No AR. (**F**) Transgastric view showing no significant central or paravalvular aortic regurgitation.

On longitudinal follow-up, including more than six months postpartum, she continued to demonstrate persistently elevated transvalvular gradients, with symptoms limited to mild exertional dyspnea during significant physical activity. The patient expressed a strong desire for additional pregnancies and wished to avoid repeated open-heart surgeries over her lifetime. She also preferred to avoid long-term anticoagulation associated with mechanical valve replacement in order to pursue future pregnancies safely, favoring a bioprosthetic strategy. Computed tomography revealed a maximum ascending aortic diameter of 4.1 cm, an aortic annular area of 3.25 cm^2^, and an annular perimeter of 65.3 mm. After extensive multidisciplinary heart team discussions incorporating the patient’s goals and long-term management considerations, she was offered transcatheter aortic valve replacement using a 26 mm Medtronic Evolut FX valve (Medtronic, Santa Ana, CA, USA). A self-expanding valve was selected primarily due to the small annular size (20 mm BEV versus 26 mm SEV), given its expected superior hemodynamic profile in a young, highly active patient who plans to have future pregnancies, which are associated with increased cardiac output and volume-related changes. The procedure was successfully performed. A staged management plan was established, with anticipation of future TAVR explantation and mechanical aortic valve replacement later, while continuing close surveillance of the aortic root in the interim (Figure 5).

**Clinical lesson:** In young patients with small annuli and anticipated long life expectancy, initial valve selection should prioritize hemodynamic performance and preservation of future reintervention options within a lifetime management framework.

## 5. Preparations for TAVR

### 5.1. TAVR in Patients with Comorbid Conditions

Chronic kidney disease is a common comorbidity among patients undergoing treatment for severe aortic stenosis. Transcatheter aortic valve replacement requires exposure to iodinated contrast during preprocedural computed tomography planning, coronary angiography, and the valve implantation itself, which increases the risk of acute kidney injury (AKI). An analysis from the National Cardiovascular Data Registry demonstrated that among patients with normal baseline renal function, those who developed stage 3 AKI had a more than sevenfold increase in adjusted 1-year mortality compared with those who did not develop AKI, with an overall AKI incidence exceeding 10% [21]. The risk of AKI is further amplified in patients presenting with cardiogenic shock who require urgent aortic valve intervention. Given the broad availability of TAVR and its increasing use in patients with elevated surgical risk, these concerns are particularly relevant.

Strategies aimed at reducing or eliminating iodinated contrast exposure are therefore highly desirable, provided they can be implemented safely. Approaches such as low-contrast computed tomography TAVR protocols, intraoperative use of multiple pigtail catheters to obtain coplanar or cusp-overlap fluoroscopic views, and the use of carbon dioxide angiography for large-bore vascular access have the potential to substantially reduce contrast utilization in high-risk patients. Emerging evidence from case reports and small observational studies suggests that zero-contrast TAVR may be feasible and associated with favorable outcomes in selected patients [22]. This strategy may be more readily applicable in valve-in-valve TAVR following prior surgical aortic valve replacement, where the risk of coronary obstruction is lower in the presence of large sinus dimensions, high coronary ostia, and clearly adequate valve-to-coronary distances.

### 5.2. Case Example

**Figure 6:**
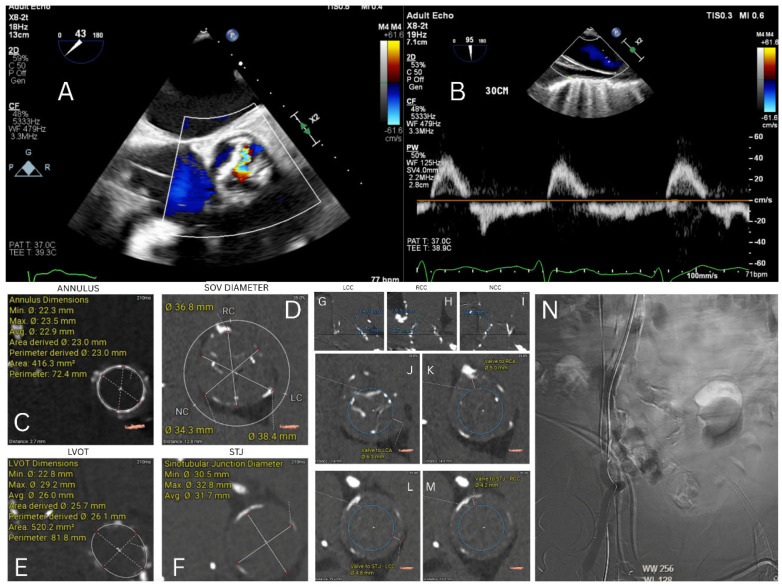
(**A**) TEE-PSAX view showing severe AI. (**B**) Diastolic flow reversal. (**C**) Annulus. (**D**) SOV Diameter. (**E**) LVOT. (**F**) STJ. (**G**–**I**) Coronary heights with corresponding STJ heights. (**J**) Valve to LCA. (**K**) Valve to RCA. (**L**) Valve to STJ-LCC. (**M**) Valve to STJ-RCC. (**N**) Post large-bore access closure peripheral CO_2_ angiography.

An 83-year-old male with a history of CKD stage IV and prior surgical aortic valve replacement using a 25 mm Trifecta valve, along with saphenous vein graft bypass to the right coronary artery 12 years earlier, presented with acute exacerbation of congestive heart failure with preserved LVEF and acute renal failure on underlying CKD. Transesophageal echocardiogram demonstrated severe aortic insufficiency with a flail right coronary leaflet and associated aortic diastolic flow reversal. Right heart catheterization revealed severely elevated cardiac filling pressures, with a mean PCWP of 46 mmHg, V wave of 64 mmHg, mean right atrial pressure of 13 mmHg, and calculated cardiac index of 2 L/min/m^2^. The calculated STS risk for mortality with redo surgical aortic valve replacement was 9.6%, with an estimated risk of renal failure of 34%. Given his elevated risk of renal injury and potential need for renal replacement therapy, non-contrast gated cardiac CT was performed for valve-in-valve TAVR planning. Intraoperatively, CO_2_ angiography was utilized for large-bore access management for transfemoral TAVR (Figure 6).

**Clinical lesson:** Zero-contrast or contrast-minimizing strategies can enable safe TAVR in high-risk renal patients when supported by careful imaging and procedural planning.

Concomitant coronary artery disease is frequently encountered in patients with severe aortic stenosis referred for TAVR, reflecting shared cardiovascular risk factors. Invasive coronary angiography remains the reference standard for diagnosing and treating obstructive coronary disease, particularly involving the major epicardial vessels. However, small observational studies have evaluated the role of computed tomography performed for TAVR planning in assessing underlying coronary artery disease and have reported acceptable safety outcomes [23]. Over time, increasing experience with transcatheter valve therapies has also led heart teams to adopt more selective thresholds for coronary revascularization, particularly in patients without angina, those with branch vessel disease, or those with unfavorable anatomical or clinical features for percutaneous coronary intervention. Collectively, these evolving practices may contribute to a reduction in contrast exposure and a lower risk of contrast-associated renal injury in the TAVR population.

## 6. TAVR in Patients with Native Annulus or Degenerated Bioprosthetic Valve Stenosis with Underlying PPM

Prosthesis–patient mismatch has been associated with an increased risk of cardiac death, heart failure-related hospitalization and repeat aortic valve intervention over time [24]. Recognition of preoperative high-risk clinical features, including female sex, in conjunction with routine use of cardiac-gated computed tomography, should be considered standard in the evaluation of all patients with aortic stenosis to identify those with smaller annular dimensions who are at increased risk for prosthesis–patient mismatch. When a small aortic annulus is identified, every effort should be made to facilitate informed decision-making aimed at minimizing the risk of mismatch through appropriate valve type and size selection and consideration of annular enlargement when a surgical approach is chosen. Such strategies may also facilitate future valve-in-valve TAVR, if required, while reducing the likelihood of significantly elevated residual transvalvular gradients.

In clinical practice, patients with severe aortic stenosis due to degeneration of bioprosthetic valves frequently present with underlying moderate to severe prosthesis–patient mismatch. Many of these patients are at increased risk for mortality and substantial morbidity with redo surgical valve replacement. Data from the EXPLANTORREDO-TAVR registry evaluating patients with failed transcatheter valves demonstrated that TAVR explantation was associated with significantly higher 30-day mortality compared with redo TAVR, with rates of 13.6% versus 3.4%, respectively (*p* < 0.001) [25]. Consequently, valve-in-valve TAVR has increasingly been adopted by heart teams as a treatment strategy for patients with failed surgical or transcatheter bioprostheses.

Techniques such as bioprosthetic valve remodeling and bioprosthetic valve fracture have been well described as methods to facilitate optimal expansion of the surgical valve frame and reduce residual transvalvular gradients following valve-in-valve TAVR [26]. In addition, higher implantation of the transcatheter valve within the failed bioprosthesis and the use of supra-annular self-expanding valves have been associated with lower residual gradients and improved hemodynamic outcomes [27]. However, substantial variability exists among operators in the application of these techniques [28]. Although anecdotal experience supports the use of valve-in-valve TAVR in patients with pre-existing prosthesis–patient mismatch, comparative studies evaluating outcomes of these strategies in this specific population are currently lacking.

## 7. TAVR in Patients with Native Aortic Stenosis or Degenerated Bioprosthetic Valve Stenosis and Risk of Coronary Obstruction: Techniques and Strategies

Coronary artery obstruction is a rare but catastrophic complication of transcatheter aortic valve replacement, occurring in approximately 0.7% of procedures and associated with mortality rates approaching 50% despite successful percutaneous or surgical rescue [29]. The risk is substantially higher in valve-in-valve TAVR, with reported incidence rates of up to 2.3% [30]. Several preventive strategies have been developed to mitigate this risk, including snorkel stenting, BASILICA (Bioprosthetic or Native Aortic Scallop Intentional Laceration to Prevent Iatrogenic Coronary Artery Obstruction), and more recently described leaflet modification and translocation techniques such as UNICORN, BICORN, and BABICORN.

Snorkel stenting is a relatively simple and widely adopted technique in which a coronary stent is prepositioned within the coronary artery at risk and deployed with protrusion into the aorta following transcatheter valve implantation to maintain coronary perfusion [31]. However, this approach carries important limitations, including the risk of stent under expansion or crushing behind the transcatheter valve frame. As a result, concerns persist regarding long-term durability, including the risks of late stent thrombosis and delayed coronary obstruction. In addition, future coronary re-access may be technically impossible following snorkel stenting, thereby limiting downstream treatment options. Despite these drawbacks, snorkel stenting remains the most commonly used coronary protection strategy in real-world TAVR practice.

Several alternative approaches have been developed that involve intentional modification of native or prosthetic valve leaflets to prevent coronary obstruction during TAVR. BASILICA was the first technique described in this category and involves traversal of an electrified guidewire through the base of the target aortic leaflet, followed by snare externalization from the left ventricular side to create a wire loop. After minor benchtop modification of the wire to concentrate electrical charge, controlled electrified laceration of the leaflet is performed along its centreline prior to transcatheter valve implantation. BASILICA has been prospectively studied and has demonstrated safety and effectiveness in preventing coronary obstruction during TAVR [32].

The ShortCut device (Pi-Cardia, Rehovot, Israel) represents the first purpose-built device designed to facilitate targeted leaflet splitting to prevent coronary obstruction. It became commercially available in January 2024 after receiving U.S. Food and Drug Administration approval based on pivotal trial data demonstrating its safety and effectiveness [33].

While centreline leaflet laceration using BASILICA allows splaying of native or surgically implanted bioprosthetic leaflets, this effect may be limited in TAVR-in-TAVR procedures due to the presence of a circumferential metallic valve frame that restricts leaflet expansion. In such cases, incomplete or absent leaflet splay may still result in coronary obstruction. Consequently, additional leaflet modification strategies involving leaflet translocation have been proposed, particularly in scenarios involving extreme leaflet overhang, very small or absent valve-to-coronary distances, or when modification of more than one leaflet is required. Although these approaches are conceptually appealing, robust safety, efficacy, and reproducibility remain limited.

The UNICORN technique [34] was developed to mitigate the risk of coronary obstruction in TAVR-in-TAVR procedures. This approach involves traversal of a radiofrequency-enabled wire through the base of the target leaflet, followed by stepwise exchange to larger guidewires and serial balloon dilation of the traversal site. A balloon-expandable transcatheter valve is then advanced through the modified leaflet and implanted in an intra-leaflet position, resulting in controlled laceration and entrapment of the leaflet between the valve frames. This design aims to prevent leaflet recoil, coronary obstruction, or embolization.

BABICORN, defined as Bileaflet Alteration with BASILICA and Iatrogenic Cusp Obliteration Using a Radiofrequency Needle, was described as a double-leaflet modification strategy for TAVR-in-SAVR procedures [35]. This technique potentially simplifies the procedure by requiring a single electrosurgical loop compared with dual BASILICA and may shorten the duration of acute aortic insufficiency associated with prolonged leaflet laceration.

**Figure 7:**
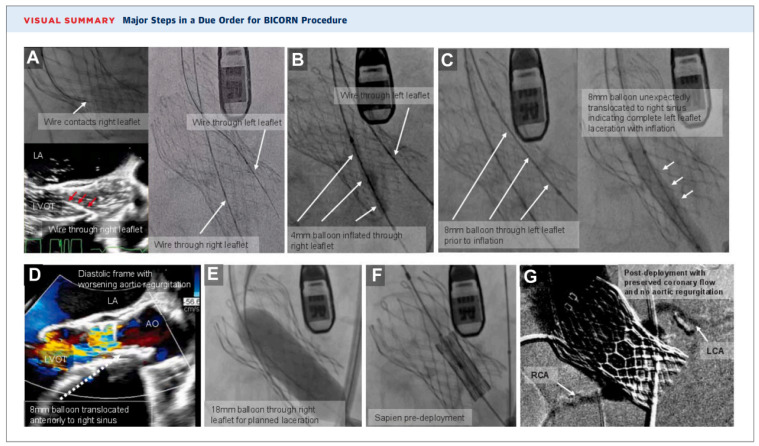
Bilateral Iatrogenic Coronary Obstruction Risk Nullification Procedure for Transcatheter Aortic Valve-in-Valve Replacement. (**A**): Electrified 0.014-in ASTATO wires were traversed through the base of the right and left coronary leaflets of the preexisting CoreValve, which were then swapped with 0.035-in wires. (**B**): A 4 mm balloon was inflated through the right leaflet. (**C**): An 8 mm balloon was advanced through the left leaflet. Upon inflation, the balloon translocated unexpectedly to the right sinus indicating complete left leaflet laceration. (**D**): There was acute worsening of aortic valve insufficiency due to unexpected left leaflet laceration. (**E**): An 18 mm True balloon was inflated through the right leaflet performing planned right leaflet laceration. (**F**): Sapien valve was deployed. (**G**): Post-deployment angiogram showing preserved coronary flow. Adapted from [36].

BICORN, or Bilateral Iatrogenic Coronary Obstruction Risk Nullification [36], was described for TAVR-in-TAVR using a balloon-expandable valve implanted within a pre-existing supra-annular self-expanding valve in patients with critically small bilateral valve-to-coronary distances. This approach seeks to prevent significant leaflet overhang from supra-annular valve leaflets that could otherwise result in recurrent valve failure. Both coronary leaflets are traversed using electrified coronary guidewires, exchanged for 0.035-inch wires, and serially dilated with progressively larger balloons. The balloon-expandable valve is then loaded through the base of the left coronary leaflet and positioned in the ascending aorta. The right coronary leaflet is translocated using an 18 mm balloon, after which the balloon and wire are removed, followed by rapid deployment of the transcatheter valve through the left coronary leaflet. While this approach may be effective in selected anatomies, it carries theoretical risks of leaflet recoil or embolization and requires further evaluation to establish its safety and efficacy (Figure 7).

**Figure 8:**
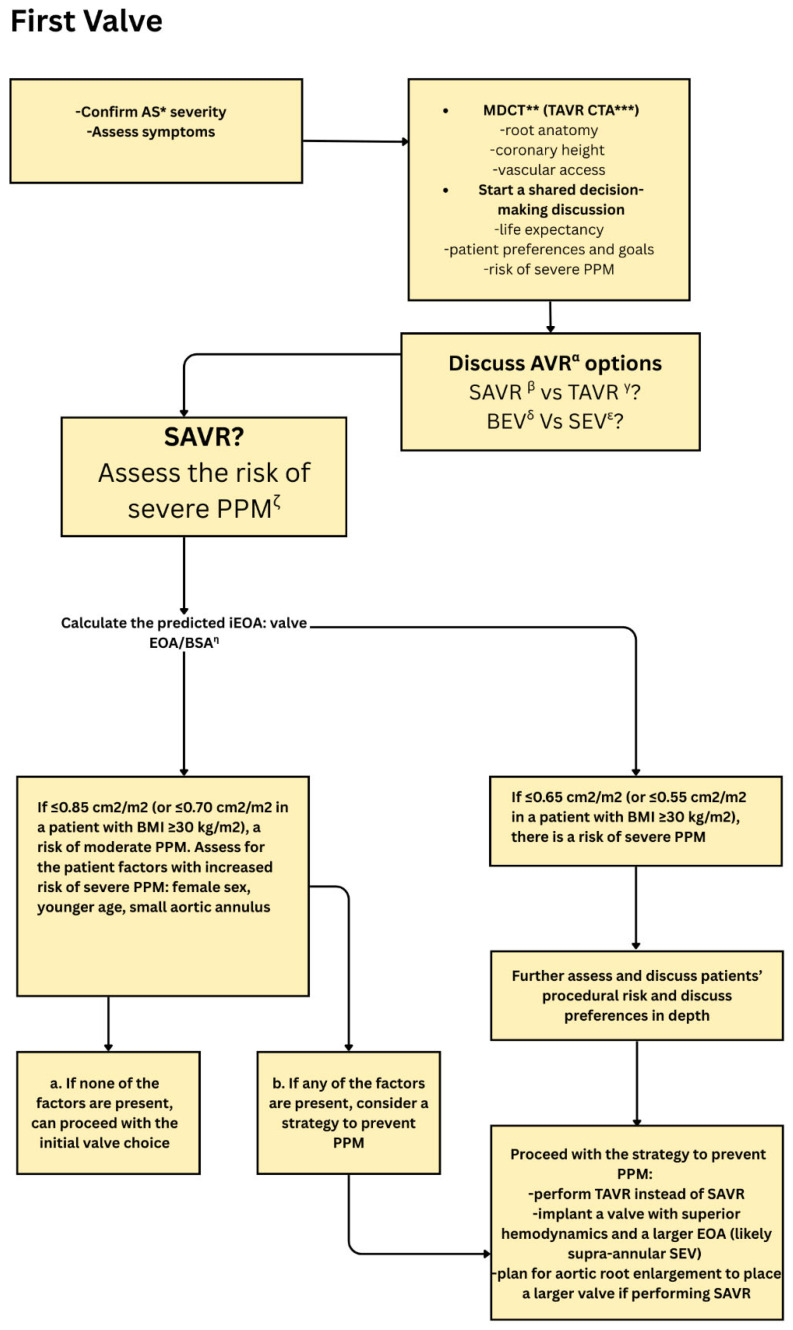
Algorithm for initial aortic valve replacement selection emphasizing confirmation of severe aortic stenosis, CT-based anatomical assessment, and shared decision-making. The figure outlines evaluation of prosthesis-patient mismatch risk using predicted iEOA, with thresholds guiding choice between SAVR and TAVR, valve type (BEV vs SEV), and strategies to mitigate moderate or severe PPM. * = Aortic stenosis, ** = Multi-detector computed tomography, *** Computed tomography angiography, α = Aortic valve replacement, β = Surgical aortic valve replacement, γ = Transcatheter aortic valve replacement, δ = Balloon-expandable valve, ε = Self-expanding valve, ζ = Patient prosthesis mismatch, η = Indexed effective orifice area: Valve effective orifice area/body surface area.

**Figure 9:**
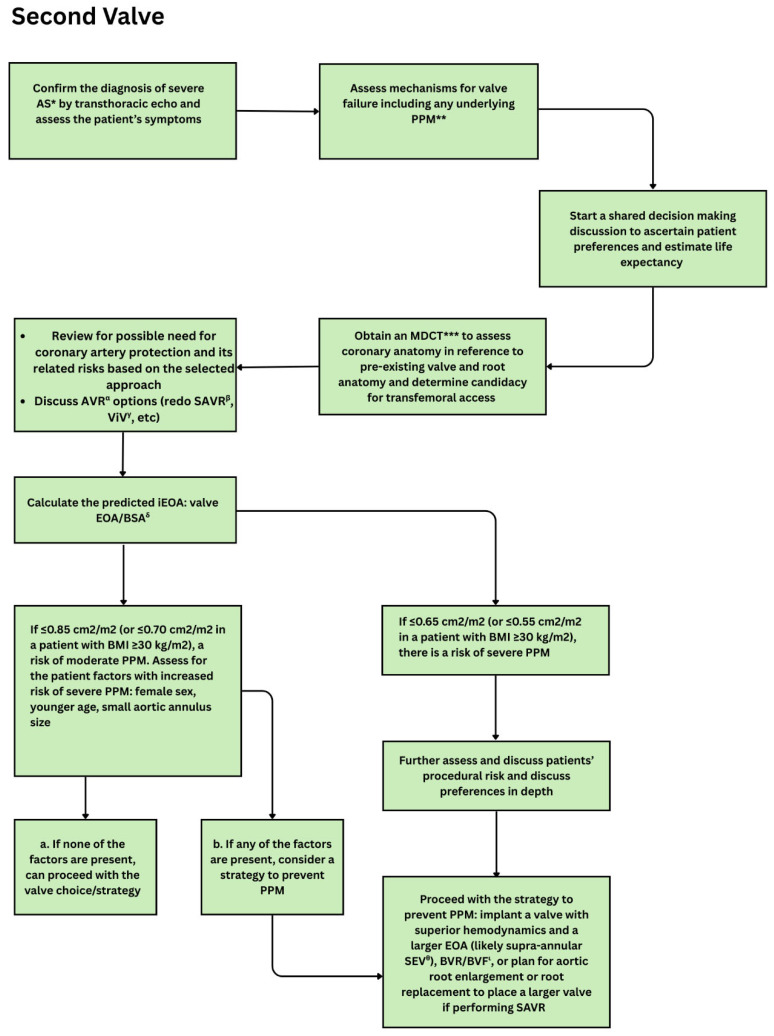
Approach to re-intervention after valve failure, incorporating mechanism of failure, coronary anatomy assessment, and shared decision-making. The figure outlines evaluation of PPM risk using predicted iEOA and guides selection among redo SAVR, valve-in-valve TAVR, and adjunctive strategies (e.g., coronary protection, bioprosthetic valve fracture) to optimize hemodynamics and prevent severe PPM. * = Aortic stenosis, ** = Patient prosthesis mismatch, *** = Multi-detector computed tomography, α = Aortic valve replacement, β = Surgical aortic valve replacement, γ = Valve in valve, δ = Indexed effective orifice area: Valve effective orifice area/body surface area, θ = Self-expanding valve, ι = Bioprosthetic valve remodeling/Bioprosthetic valve fracture.

Although many techniques have been described to help prevent coronary obstruction during TAVR in patients with high-risk CT features, some of which are currently supported only by case reports or small series (e.g., UNICORN, BICORN, BABICORN), meticulous evaluation is required to safely select patients for their application. This becomes even more important in cases of sinus misalignment or malalignment, near-zero valve-to-coronary distance, and calcified coronary take-offs at risk (Figure 8 and Figure 9).

## 8. TAVR in Patients with Concomitant Prosthesis–Patient Mismatch and Coronary Obstruction Risk

Externally mounted leaflet surgical aortic valves are designed to achieve larger effective orifice areas and improved hemodynamic profiles and are therefore more commonly implanted in patients with smaller aortic annuli. As a result, these patients may be at increased risk of coronary obstruction during valve-in-valve TAVR, particularly when valve-to-coronary distances are small, coronary heights are low, and sinus dimensions are limited. The Trifecta valve is a representative example of an externally mounted leaflet valve and was withdrawn from the U.S. market because of concerns regarding early structural valve degeneration requiring repeat valve intervention [9].

Prior studies have also demonstrated a higher incidence of prosthesis–patient mismatch following surgical aortic valve replacement compared with TAVR. Consequently, the coexistence of prosthesis–patient mismatch and elevated coronary obstruction risk is not uncommon in contemporary practice. In such scenarios, strategies combining leaflet modification with bioprosthetic valve remodeling or bioprosthetic valve fracture may be employed to mitigate both residual transvalvular gradients and the risk of coronary obstruction. However, data evaluating the safety, efficacy, and long-term outcomes of these combined approaches in patients with concomitant prosthesis–patient mismatch and coronary obstruction risk remain limited.

## 9. Future Directions

### 9.1. Indications

Transcatheter aortic valve replacement has become a well-established therapeutic option for patients with severe aortic stenosis and now includes asymptomatic patients following U.S. Food and Drug Administration approval on 1 May 2025, based on the results of the EARLY TAVR trial [1,2]. In parallel, currently available transcatheter heart valves are being used off label in select inoperable patients with severe native aortic insufficiency, although this approach is associated with higher risks of device embolization, reported in approximately 15% of cases, and the need for a second valve, reported in approximately 11% [37].

The Trilogy valve (JenaValve Technology, Irvine, CA, USA), a dedicated transcatheter heart valve designed for patients with moderate to severe or severe aortic insufficiency who are at high surgical risk, demonstrated favorable safety outcomes in the ALIGN-AR trial [38]. This device employs a clip-based anchoring mechanism that secures the prosthesis to the native valve leaflets and does not rely on annular calcification or radial force for stability.

Interest in the potential benefits of TAVR in patients with moderate aortic stenosis remains high. However, the TAVR UNLOAD trial [33], which compared transfemoral TAVR vs. clinical surveillance in patients with moderate aortic stenosis and heart failure with reduced ejection fraction (HFrEF) on optimal guideline-directed medical therapy (GDMT), was terminated early after enrolling 178 of the planned 600 participants and did not demonstrate a significant benefit in the composite endpoint of death, disabling stroke, heart failure hospitalization, or quality of life at one year. Ongoing randomized trials continue to evaluate this population, including the EXPAND TAVR II pivotal trial and the PROGRESS trial, the latter of which focuses on patients with moderate aortic stenosis with symptoms or evidence of cardiac damage/dysfunction. Results from these studies may further inform patient selection and potentially lead to additional expansion of TAVR indications in the future.

### 9.2. Device and Delivery System Upgrades

Continuous innovation by industry has led to significant advancements in transcatheter heart valve devices and delivery systems. The SAPIEN 3 Ultra RESILIA valve (Edwards Lifesciences, Irvine, CA, USA) represents the latest iteration of the balloon-expandable SAPIEN platform and incorporates several refinements over prior generations. These include an enhanced sealing skirt and leaflet suspension mechanisms designed to further reduce paravalvular regurgitation, as well as calcium-blocking tissue processing of bovine pericardial leaflets aimed at delaying structural valve deterioration.

Similarly, the Evolut FX delivery system (Medtronic) features a redesigned nose cone and increased capsule flexibility to facilitate navigation through challenging vascular anatomies. The addition of a single-spine shaft further enhances flexibility and deliverability. The Evolut FX+ valve incorporates larger and more conspicuous radiopaque markers to improve visualization of deployment depth and commissural orientation, as well as two enlarged cells designed to be aligned with the coronary ostia, potentially facilitating future coronary access.

Several next-generation transcatheter valves are also emerging. The MiRus Siegel valve, evaluated in a U.S. Early Feasibility Study presented in July 2025, introduces multiple novel features for the treatment of symptomatic aortic stenosis. These include an 8-French delivery sheath that may enable less invasive procedures and expand patient eligibility, a nickel-free platform composed of proprietary rhenium alloys suitable for patients with nickel hypersensitivity, precise deployment without foreshortening, intrinsic commissural alignment, and pre-mounted dry porcine pericardial leaflets treated with anti-calcification technology.

The DurAVR transcatheter heart valve (Anteris Technologies, Eagan, MN, USA) is another balloon-expandable system featuring a supra-annular leaflet design intended to optimize hemodynamic performance. This valve employs a biomimetic architecture created from a single piece of proprietary ADAPT tissue molded into native-shaped leaflets and includes wide open cells to facilitate future coronary access. Following U.S. Food and Drug Administration approval in November 2025, the DurAVR valve is scheduled to enter the PARADIGM global investigational device exemption trial.

## 10. Leaflet Modification Tools

Modification of native or pre-existing bioprosthetic aortic valve leaflets has become a critical component of lifetime valve management in patients undergoing TAVR who would otherwise face prohibitive risk of coronary artery obstruction. While electrified coronary guidewires remain widely used for leaflet traversal and laceration, dedicated systems have now been developed to streamline and standardize these procedures.

The Transmural Electrosurgery Leaflet Traversal and Laceration Evaluation BASILICA-TAVR system represents the first purpose-built platform for leaflet laceration and includes an insulated guidewire, shaped guide catheters, and accessory components designed to improve procedural efficiency and safety [39]. The ShortCut device (Pi-Cardia) is the first dedicated mechanical system designed to split bioprosthetic valve leaflets during valve-in-valve TAVR without the use of electrosurgical energy, potentially enhancing procedural safety and ease of use [40].

In certain anatomical scenarios, leaflet laceration alone may be insufficient to prevent acute or delayed coronary obstruction, particularly in cases involving prior high TAVR implantation, lack of commissural alignment, or supra-annular leaflet designs that necessitate higher valve-in-valve deployment. In these settings, techniques incorporating leaflet translocation, such as UNICORN and BICORN, have been described.

The Catheter Electrosurgical Debulking and Removal procedure (CATHEDRAL) involves excision of a bioprosthetic leaflet using an electrified guidewire to secure the leaflet in combination with an electrified rotatable polypectomy snare to detach the tissue [41]. Although initially described for removal of flail leaflets, this approach may be adapted for excision of intact leaflets as operator experience and technical proficiency evolve. With growing procedural demand, it is anticipated that dedicated devices will be developed to further enhance the safety and reproducibility of leaflet excision techniques.

## 11. Advanced Imaging Tools: Simulation-Based Procedural Planning

Computed tomography-based computer modeling remains the cornerstone of preprocedural planning for TAVR, with platforms such as 3mensio commonly used in clinical practice. More recently, advanced simulation-based imaging tools have emerged, including finite element and computational modeling systems such as FEOPS, DASI, and Ansys [42]. These platforms offer enhanced insight into anatomical interactions between native tissues and transcatheter valves beyond conventional sizing.

In addition to assisting with valve selection, these tools have the potential to predict complications such as annular rupture, paravalvular regurgitation, conduction disturbances, and leaflet thrombosis. However, large prospective studies are needed to validate their accuracy, reproducibility, and impact on procedural and long-term clinical outcomes before widespread adoption can be recommended.

As TAVR expands into younger and more complex patient populations, device-related management has become increasingly important. Conduction disturbances, including new-onset left bundle branch block and high-grade atrioventricular block requiring permanent pacemaker implantation, remain among the most common complications following TAVR. These complications are closely linked to procedural factors such as implantation depth, valve type, and membranous septum anatomy. Preprocedural planning and intraprocedural techniques aimed at minimizing conduction system injury are therefore critical. In addition, long-term management of cardiac implantable electronic devices, including infection prevention, device surveillance, and lead-related considerations, is increasingly relevant in this population. These complications have important implications for length of stay, rehospitalization, and long-term outcomes. As structural and electrophysiological care increasingly intersect, a multidisciplinary approach to device management is essential for optimizing long-term outcomes [43].

Artificial Intelligence and machine learning have significantly improved the processes of patient selection and safe delivery of care by predicting possible periprocedural complications for appropriate planning for safety measures. These tools have allowed the standardization of diagnostic testing-based assessments, rapid and timely recognition of abnormal findings and streamlining of patient care pathways.

Beyond simulation-based modeling, emerging visualization technologies such as holographic and mixed-reality platforms are expanding the scope of procedural planning [44]. These tools enable three-dimensional, patient-specific visualization of complex aortic root anatomy and device–tissue interaction. This may be particularly valuable in anatomically challenging cases, including bicuspid aortic valves, low coronary heights, and valve-in-valve procedures. Early experience suggests that these technologies may enhance spatial understanding, procedural strategy, and team-based decision-making. However, their integration into routine clinical practice remains in early stages and requires further validation.

## 12. Limitations

This review has several limitations. First, as a narrative review, the article does not follow a formal systematic methodology and is therefore subject to selection bias. Although efforts were made to include high-quality and clinically relevant studies, the inclusion of literature was not exhaustive, and some important studies may not have been captured.

Second, the field of transcatheter aortic valve replacement is rapidly evolving, with ongoing trials and emerging technologies that may alter current understanding and practice. As such, some of the data discussed, particularly regarding newer devices and techniques, may become outdated as additional evidence becomes available.

Third, several of the advanced procedural strategies discussed, including leaflet modification techniques such as BASILICA, ShortCut, UNICORN, BICORN, and related approaches, are supported primarily by case reports, small series, or early feasibility studies. Robust long-term outcomes and comparative data remain limited, which restricts the ability to draw definitive conclusions regarding their safety, durability, and generalizability.

Finally, heterogeneity across studies in terms of patient populations, valve platforms, imaging protocols, and outcome definitions limits direct comparison between trials and may influence the interpretation of reported outcomes. These limitations should be considered when applying the findings of this review to clinical practice.

## 13. Conclusions

Since its introduction, transcatheter aortic valve replacement has undergone remarkable evolution, driven by advances in device design, procedural techniques, patient selection, and expanding indications. These developments have improved outcomes and broadened access but have also increased the complexity of procedural planning and device selection. Successful contemporary TAVR practice requires a comprehensive understanding of valve technologies, procedural strategies, and anatomical considerations, supported by a multidisciplinary heart team and a continuous learning environment.

First valve selection remains the most critical step in the lifetime management of aortic valve disease and should incorporate careful planning, anticipation of future interventions, and thorough patient education. Optimal valve choice is inherently multifactorial and influenced by anatomical constraints, hemodynamic and durability expectations, and long-term management strategies that may include subsequent valve-in-valve therapy. When reintervention becomes necessary, meticulous valve-in-valve selection and procedural planning represent the next pivotal step to ensure durable hemodynamic performance and patient safety.

Despite these advances, it is important to recognize that long-term outcomes of TAVR in younger patients remain limited, and surgical aortic valve replacement continues to have more established durability data in this population. Accordingly, careful patient selection and individualized decision-making remain essential when considering TAVR in younger, low-risk patients.

## Data Availability

No new data were created or analyzed in this study. Data sharing is not applicable to this article.

## Abbreviations

AKI	Acute kidney injury
AR	Aortic regurgitation
AS	Aortic stenosis
AV	Aortic valve
AVA	Aortic valve area
AVR	Aortic valve replacement
BASILICA	Bioprosthetic or Native Aortic Scallop Intentional Laceration to Prevent Iatrogenic Coronary Artery Obstruction
BICORN	Bilateral Iatrogenic Coronary Obstruction Risk Nullification
BVF	Bioprosthetic valve fracture
BVR	Bioprosthetic valve remodeling
CKD	Chronic kidney disease
CT	Computed tomography
EOA	Effective orifice area
FDA	U.S. Food and Drug Administration
GDMT	Guideline-directed medical therapy
iEOA	Indexed effective orifice area
LCA	Left coronary artery
LCC	Left coronary cusp
LVEF	Left ventricular ejection fraction
LVOT	Left ventricular outflow tract
NCC	Non-coronary cusp
PPM	Prosthesis–patient mismatch
PVL	Paravalvular leak
RCA	Right coronary artery
RCC	Right coronary cusp
RCT	Randomized controlled trial
SAVR	Surgical aortic valve replacement
SOV	Sinus of Valsalva
STS	Society of Thoracic Surgeons
STJ	Sinotubular junction
TAVI	Transcatheter aortic valve implantation
TAVR	Transcatheter aortic valve replacement
TEE	Transesophageal echocardiography
TTE	Transthoracic echocardiography
TVT	Transcatheter Valve Therapy
